# Factors associated with outcomes for looked-after children and young people: a correlates review of the literature

**DOI:** 10.1111/j.1365-2214.2011.01226.x

**Published:** 2011-09

**Authors:** R Jones, E S Everson-Hock, D Papaioannou, L Guillaume, E Goyder, J Chilcott, J Cooke, N Payne, A Duenas, L M Sheppard, C Swann

**Affiliations:** * School of Health and Related Research, The University of SheffieldSheffield; † National Institute for Health and Clinical ExcellenceManchester, and; ‡ National Institute for Health and Clinical ExcellenceLondon, UK

**Keywords:** behavioural problems, correlates, emotional well-being, looked-after children and young people, outcomes, placement stability

## Abstract

In 2008, the Department of Health made a referral to the National Institute for Health and Clinical Excellence and the Social Care Institute for Excellence to develop joint public health guidance on improving the physical and emotional health and well-being of children and young people looked after by the local authority/state. To help inform the decision-making process by identifying potential research questions pertinent to the outcomes of looked-after children and young people (LACYP), a correlates review was undertaken. Iterative searches of health and social science databases were undertaken; searches of reference lists and citation searches were conducted and all included studies were critically appraised. The correlates review is a mapping review conducted using systematic and transparent methodology. Interventions and factors that are associated (or correlated) with outcomes for LACYP were identified and presented as conceptual maps. This review maps the breadth (rather than depth) of the evidence and represents an attempt to use the existing evidence base to map associations between potential risk factors, protective factors, interventions and outcomes for LACYP. Ninety-two studies were included: four systematic reviews, five non-systematic reviews, eight randomized controlled trials, 66 cohort studies and nine cross-sectional studies. The conceptual maps provide an overview of the key relationships addressed in the current literature, in particular, placement stability and emotional and behavioural factors in mediating outcomes. From the maps, there appear to be some key factors that are associated with a range of outcomes, in particular, number of placements, behavioural problems and age at first placement. Placement stability seems to be a key mediator of directional associations. The correlates review identified key areas where sufficient evidence to conduct a systematic review might exist. These were: transition support, training and support for carers and access to services.

## Introduction

Looked-after children and young people (LACYP) in the UK are children and young people in the care of the local authority, either voluntarily or subject to a care order made by court to grant shared parental responsibility with a local authority (‘in care’). There were approximately 60 000 LACYP in England at 31 March 2007 (Department for Children, Schools and Families 2008). They are looked after in a range of different settings. While 71% of LACYP were in a foster placement in 2007, others are placed in a variety of types of setting including residential homes (10%), residential schools (2%) or secure unit settings (Department for Children, Schools and Families 2008).

Children and young people are placed outside their parents' care for many reasons. These include physical abuse, sexual abuse, emotional or psychological abuse, various types of neglect and other circumstances which prevent parents from adequately caring for their children. LACYP will have been exposed to multiple risks associated with poor long-term outcomes before entering care. Entering care is also strongly associated with poverty and deprivation including low income, parental unemployment and relationship breakdown, and over 60% of children are in care because of abuse or neglect (Department for Children, Schools and Families 2008). LACYP are also more likely to experience educational, behavioural, physical and psychological problems (Meltzer *et al*. 2003). Using data from a 1970 British birth cohort, one study reported on a cohort of children that had been in care (Viner & Taylor 2005). The report found that being in care during childhood is associated with adverse adult socio-economic, educational, legal and health outcomes in excess of that associated with coexisting childhood or adult disadvantage.

A group of particular concern, but for whom little data are available, are unaccompanied asylum-seeking children (UASC) who are referred to local social services departments for support and now make up approximately 10% of all those in local authority care and represent over 50% of the care population in some local authority areas (Youth Justice Board, http://www.yjb.gov.uk/en-gb/practitioners/Diversity/Refugees/). UASC not only need protection from direct discrimination and racism, they also need special consideration from all agencies involved in their care to ensure that their social, educational, psychological and health needs are appropriately assessed and met.

### Purpose of the review

The researchers carried out a correlates review on LACYP on behalf of the National Institute for Health and Clinical Excellence (NICE) and the Social Care Institute for Excellence (SCIE) to develop joint public health guidance on improving the physical and emotional health outcomes for LACYP. Correlation is a measure of the linear relationship (or the association) between two variables; for a correlates review, the variables are factors and outcomes. The review used an iterative searching technique where a number of iterations were undertaken to search for evidence using freetext and keyword/index term searching of databases. It was decided that the best way to present the evidence was to use conceptual maps linked to evidence tables. Full details of the search strategies, methods and evidence tables are available from the full report informing the NICE guidance (Jones *et al*. 2010).

The aim of the review was to identify factors that are associated with outcomes for LACYP, and present this evidence as conceptual maps, giving an indication of the types of reported studies on LACYP and the interventions, factors and outcomes reported. This is likely to assist in identifying possible interventions to improve outcomes for LACYP. Factors have been included that have been evaluated in terms of their association (whether potentially causal or not) with positive or negative outcomes for LACYP. The conceptual maps, indicating possible relationships between risk factors/protective factors, interventions and outcomes for looked-after children, address the following key questions:

**Question 1:** What evidence is available on the factors (risk factors and protective factors) that are associated with short-term and long-term outcomes (positive and negative) for looked-after children and young people?
**Question 2:** What interventions, strategies or activities to improve outcomes for looked-after children and young people or their carers have been studied?
**Question 3:** What are the identified predictive factors associated with positive and negative outcomes for looked-after children and young people?
**Question 4:** What are the relationships and/or interactions between interventions, predictive factors and outcomes for looked-after children?

The review includes randomized controlled trials, cross-sectional surveys, cohort and case–control studies in which the population of interest are LACYP up to the age of 25 years and the control group is also LACYP. The exposures measured are factors hypothesized to predict outcomes of significance to individuals, family or the wider community (whether or not a causal relationship is assumed) and in which the outcomes measured may include social, health, economic, educational and behavioural outcomes.

## Methods

### Searching for the review

The aim of the search process for the review was to capture the breadth (rather than depth) of literature on factors associated with outcomes for LACYP. Once an outcome or factor had been identified, further searches and sifting sought to identify new factors/outcomes rather than identify all the evidence for each relationship depicted on the conceptual map. A number of iterations were undertaken to search for evidence for the review using freetext and keyword/index term searching of databases (see Jones *et al*. 2010). A thorough audit trail of this process was maintained, with all searches, number of hits and number of relevant references identified recorded, in order that searches were transparent, systematic and replicable. Full details of the search strategy, software used and quality assessment of included studies are available from the full report informing the NICE guidance (Jones *et al*. 2010).

### Study quality

Study quality was assessed using the checklists and guidance provided in the NICE Centre for Public Health Excellence Methods Manual (National Institute for Health and Clinical Excellence 2006), which assesses studies according to various aspects of design, sampling, measurement, analysis and reporting. Greater consideration was given to the performance of the study on criteria fundamental to the robustness of the findings. Study quality did not determine inclusion into or exclusion from the review. Studies were graded with ++, + or −, where ++ indicates that all or most of the criteria have been fulfilled and where they have not been fulfilled the conclusions are thought very unlikely to alter; + indicates that some of the criteria have been fulfilled and those that have not been fulfilled are unlikely to alter the conclusions; and – indicates that few or no criteria have been fulfilled and the conclusions of the study are thought likely or very likely to alter. Study quality was assessed by both reviewers; any disagreements on study quality were to be resolved by discussion with reference to third party in the event of no resolution. There was no disagreement on the grading of studies.

### Inclusion and exclusion criteria

The following inclusion criteria were applied to retrieved citations in order to identify relevant studies for inclusion. Exclusion was undertaken initially at title and/or abstract and then full paper level. No date limits were specified for study inclusion.

#### Population

The population under consideration was LACYP aged up to 25 years. This included retrospective or cross-sectional studies with study populations of adults who were LACYP if explanatory factors in childhood were collected.

#### Explanatory factors

1. Risk factors – Any factor that is potentially or actually associated with an increased risk of adverse outcomes or a decreased chance of beneficial outcomes.
2. Protective factors – Any factor that is potentially or actually associated with a decreased risk of adverse outcomes or an increased risk of beneficial outcomes.
3. Interventions – Any intervention or activity that affects these outcomes.

#### Outcomes

Outcomes are described as defined by individual studies. Some variables treated as risk factors in some studies may be intermediate outcomes in others. Relevant outcome measures have included behavioural problems, number of placements, placement stability, mental health, risk-taking behaviour, employment, etc. and are fully described in the draft scope and review protocol.

#### Other

Only English language papers have been included.

#### Data extraction and data synthesis

Initially, relevant information was extracted using extraction tables and papers classified according to both study type and the main variables analysed. Because of the great variety of variables and methods drawn from diverse samples, it was not possible to conduct a meta-analytical review; therefore, a conceptual map indicating the potential relationship between factors, interventions and outcomes was used.

## Results

A total of 96 studies were identified as meeting inclusion criteria: four systematic reviews, six non-systematic reviews, nine randomized controlled trials, 68 cohort studies and nine cross-sectional studies. Full details of all included studies, including a quality assessment, have been described in the full report. The vast majority of studies identified were studies carried out in a US setting and some of the US studies used the terms specific to the US care system including ‘out-of-home care’, and ‘group homes’.

### Conceptual maps

**Figure 1:**
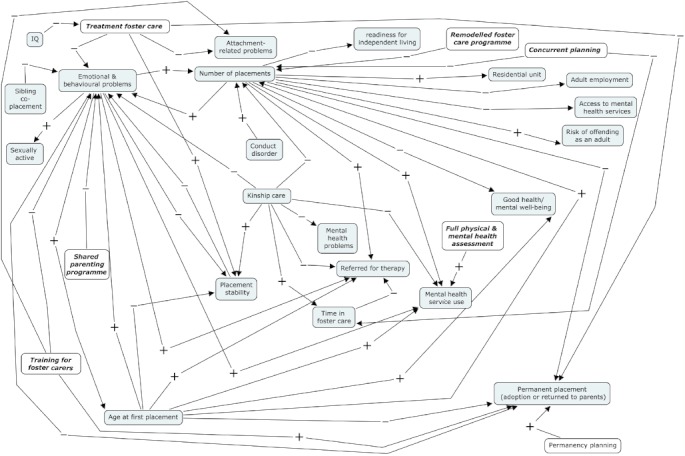
Interventions, factors and outcomes, with most reported associations. For each concept, the direction of the association is given as positive (+) or negative (−). Bold text indicates a defined intervention.

The conceptual maps display each concept, whether an intervention, factor or outcome, from the identified studies, and the association between each concept. For each concept, the direction of the association is given as positive (+) or negative (−). The direction of arrows is purely based on individual study definitions of explanatory (or independent) and outcome (or dependant) variables, with the arrow going from explanatory variable to outcome variable. For example, in Fig. 1, using the concepts ‘behavioural problems’ and ‘referred for therapy’, a positive association (+) indicates that the more behavioural problems a child has, the more likely it is that the child is referred for therapy. Similarly, a negative (−) association indicates that, for example, age at first placement and permanent placement (defined as returned to own home or permanently placed with another family, an adoptive family or foster family) indicates that the older a child is, the less likely they are to achieve a permanent placement. However, the maps only indicate the direction of the association and do not imply causality. The layout of the interventions, factors and outcomes in the conceptual maps does not imply any hierarchy of evidence or importance, but is merely for visual tidiness. Where an intervention was defined, these have been shown in bold text on the maps to aid identification.

Multiple maps were produced because of the number of associations reported by the included studies, as including all the associations on one map would have made the map difficult to read. Full details of the references to each association displayed on the maps are available from the full report informing the NICE guidance (Jones *et al*. 2010). The map in Fig. 1 shows the factors and outcomes most often reported, and there is some crossover between the conceptual maps.

**Figure 2:**
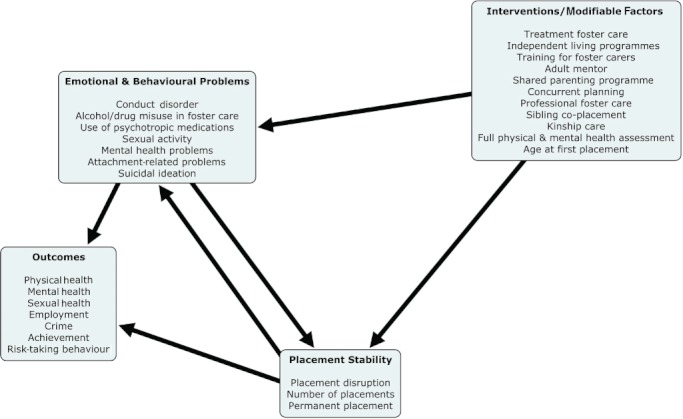
Overview, showing key associations between interventions/modifiable factors and outcomes.

**Figure 3:**
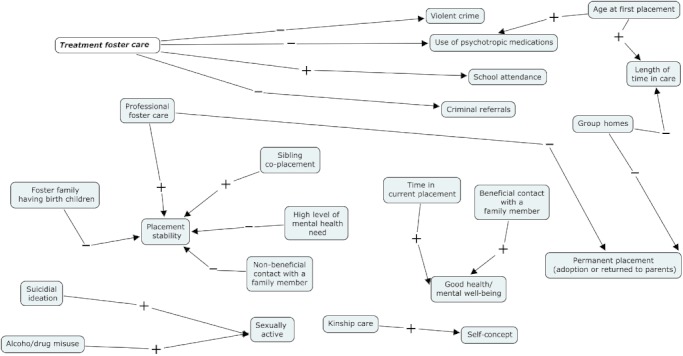
Interventions, factors and outcomes, with fewer reported associations. For each concept, the direction of the association is given as positive (+) or negative (−). Bold text indicates a defined intervention.

**Figure 4:**
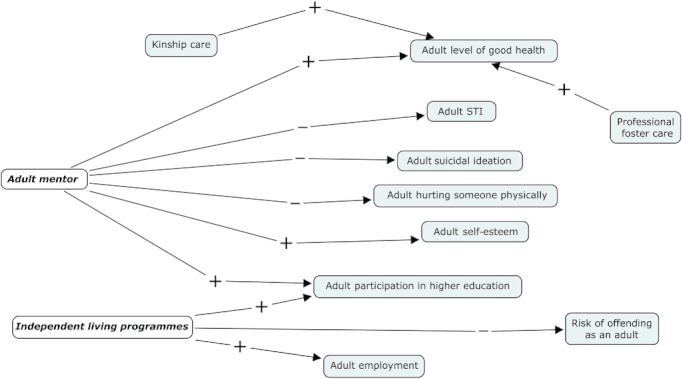
Interventions associated with adult outcomes. For each concept, the direction of the association is given as positive (+) or negative (−). Bold text indicates a defined intervention. STI, sexually transmitted infections.

Maps in Figs 1–4 were constructed first by a direct mapping of the associations reported in the published studies. The concepts within the maps were then investigated for possible grouping and classification. Analysis of these maps suggested that most factors could be grouped under the headings in Fig. 2. Both behavioural problems and placement stability feature as both factors and outcomes depending on a study's perspective. It also became apparent that interventions along with factors that could be modified could be grouped together. Finally, although a wide range of outcome measures were reported, they could be largely classified by the type of outcome they relate to as shown in Fig. 2.

### Analysis of maps

**Figure 5:**
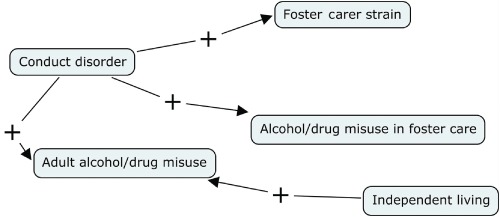
Outcomes associated with conduct disorder or independent living. For each concept, the direction of the association is given as positive (+) or negative (−).

Figure 2 displays an overview of the associations found. The majority of associations can be classified as linking interventions/modifiable factors, behavioural problems and placement stability to outcomes. Figure 5 shows how these groups are associated with each other and with reported outcomes.

The maps help identify potential direct relationships and indirect relationships. For example, in Fig. 1, one study implies that there is an association between the behavioural problems of a foster child and a referral for therapy (Cantos *et al*. 1996). Although no study was identified that showed a direct association between training for foster carers and therapy referral, another study suggested that training for foster carers would reduce foster children's behavioural problems, therefore, an indirect link could be identified using the conceptual map (Fig. 3; Chamberlain *et al*. 2008).

While one study identified behavioural problems that may potentially have an influence on the number of placements (Pardeck 1983), another study suggested that the number of placements may influence behavioural problems (Newton *et al*. 2000). Therefore, despite the references to ‘outcomes’ in study descriptions, the associations used in the conceptual map do not imply causality. Definition of ‘explanatory’ and ‘outcome’ variables may be arbitrary, where associations are not causal or where causality may operate in two directions (for example, behavioural problems and placement stability potentially both influencing each other).

Figures 3–5 display additional relationships that have little or no identified associations with the main factors and outcomes in Fig. 1. This may be either because of limited research on these factors and outcomes, or because studies linking these factors and outcomes to those in the main correlates map were not identified.

From the maps, there appear to be some key factors that are associated with a range of outcomes, in particular, number of placements, behavioural problems and age at first placement. Placement stability seems to be an outcome associated with a range of factors. The interventions most commonly described in the identified literature are treatment foster care and training for foster carers.

While associations have been mapped, some studies reported where there did not appear to be any statistically significant associations (see the full report informing the NICE guidance for details; Jones *et al*. 2010). For example, one study reported that no association was found between kinship care and participation in higher education, age at first sexual intercourse, age at first pregnancy and number of sexual partners (Carpenter *et al*. 2001). Another study reported that no association was found between kinship care and alcohol/drug misuse while in care (Usher *et al*. 1999), while a further study reported that no association was found between mental age and being referred for therapy (Cantos *et al*. 1996).

One commonly studied factor included in Fig. 1, and reported mainly in US studies, is kinship care, which has been associated with a number of outcomes for LACYP. There would seem to be a number of potential unreported benefits for the child being placed in kinship care, for example, living in the same area, attending the same school, reduced loss of separation from family, etc. However, all the studies are observational studies and no randomized controlled trials were identified. Since the correlates review, there has been more recent work carried out which adds to the evidence on kinship care (Winokur *et al*. 2009). This is conceivable as randomly allocating children to kinship care or foster care for the purposes of a trial would be ethically questionable; however, the available evidence from observational studies must be interpreted with consideration of the greater risk of bias impacting on the findings. For example, children and young people removed from their birth parents would only be placed with responsible family members that posed no risk to the child or young person and this implies selection bias in deciding whether a child or young person goes into kinship or foster care. This illustrates a more general issue that in observational studies comparing different types or setting of care, it is unlikely that the groups of children compared will have the same level of baseline risk for positive or negative outcomes, as the background and characteristics of the child will influence choice of appropriate placement.

Note also that the widely used term ‘placement stability’ that appears in the maps is defined differently by different studies. Several authors use the term as the disruption of or breakdown of the current placement (James 2004; James *et al*. 2004; Sallnas *et al*. 2004; Chamberlain *et al*. 2006; Oosterman 2007; Turner *et al*. 2007). One author uses the term ‘placement permanency’ for LACYP remaining in the same placement, which for this report is classified as placement stability (Reddy & Pfeiffer 1997). Some authors examined it in the context of number of placement moves (Pardeck 1983; Webster *et al*. 2000; Unrau *et al*. 2004), while others examined it in the context of placement patterns (James *et al*. 2004).

### Interventions or modifiable factors

The mapping exercise demonstrates that placement stability and emotional and behavioural problems are key concepts in determining outcomes for LACYP. This section focuses on the interventions and modifiable factors that have been identified that impact on placement stability and emotional or behavioural problems.

From Fig. 1, the interventions and modifiable factors can be broadly grouped under three headings: placement choice, additional service or quality of placement. The heading, ‘placement choice’, covers options that may be available to social workers at the time of placement. Age at first placement may not seem to be a modifiable factor; however, as several studies have indicated that the older the child is when first taken into care, the more likelihood there is of placement breakdown or behavioural problems, the decision of when to take a child into care or put support into the family is always something that needs to be considered (Cooper *et al*. 1987; Horwitz *et al*. 1994; Webster *et al*. 2000; James 2004; Stovall-McClough & Dozier 2004; Wilson 2004; Oosterman 2007; Shechory & Sommerfeld 2007). The heading, ‘additional services’, suggests an enhancement to normal service provision, and the heading, ‘quality of placement’, refers to interventions that aim to improve the quality of care.

At the time of a planned entry into care or a planned move, the social worker may have a range of options to choose from (subject to availability), to help decide on the most appropriate placement. Obviously, when an emergency placement is made, the options available are severely limited, but can still be considered once a planning meeting has been arranged. In this review, we called the first category ‘placement choice’. Placement choice included interventions and modifiable factors such as treatment foster care, professional foster care, sibling co-placement, kinship care and age at first placement. As well as general foster care, there are interventions that can be classed as ‘extra’ to the care given to LACYP. These interventions may be used to enhance the care for selected LACYP, which in our review were classed as additional services; these services will generally be focused on the child or young person, and the interventions and modifiable factors grouped under this heading were: independent living programmes, adult mentor, concurrent planning, transitional planning and shared parenting programme. The third category, quality of placement, covered those interventions that improve the quality of the placement and may be more focused on the carer, rather than the child. This included training for foster carers and full physical and mental health assessment.

## Discussion

This review has identified quantitative research for a range of factors that are associated with outcomes for LACYP. We have mapped the associations between factors, interventions and outcomes in an effort to produce a better understanding of the complex relationships between a range of factors and short- and long-term outcomes. It is hoped that the results of this review will promote discussion and help to identify where the priorities and main opportunities for improving outcomes for LACYP may lie. The findings identify relationships supported by evidence but do not enable us to quantify the relationships identified without further reviews to comprehensively synthesize all the evidence for any single association.

### Searching issues

The literature searching for this review was undertaken over seven iterations, using a number of sources from a range of subject areas including medical, education, social science and social service databases. While the search approach was comprehensive, it could be extended to include further identification of useful search terms from the evidence already identified and so further iterations.

### Gaps in the evidence identified

The overall findings highlight both the breadth of available evidence and some areas where there are likely to be gaps in the evidence base for potential areas for intervention. For example, there is a wealth of research in this field examining the relationships between system factors (e.g. the type, setting or organization of care) or carer-related factors (e.g. training or support) and outcomes for children, particularly in terms of the impact on placement stability and behaviour as highlighted by Fig. 1. Fewer studies examine the impact of factors or interventions that relate more directly to an intervention or modifiable factor for an individual child or young person (e.g. provision of adult mentors) or on the relationship of such interventions to adult outcomes.

Some outcomes that are clearly important because they are significantly more common among LACYP are not well represented, because the studies of these outcomes did not also identify associated factors and this did not meet the review criteria. Examples include studies on the school performance of LACYP with a physical or mental disability (Geenen & Powers 2006) and studies examining the risk of teenage pregnancy (which tend to compare LACYP with non-LACYP, rather than identifying predictive factors for LACYP).

Only a minority of studies included the full breadth of the population of LACYP. These included UK surveys such as the survey of mental health of LACYP (Meltzer *et al*. 2003). Most studies in this review have focused on specific subgroups of LACYP, particularly those in foster care (Vaughn *et al*. 2007; Macdonald & Turner 2008). There are other groups for which there appears to be virtually no specific evidence including those about which there may be particular policy concern, such as UASC or LACYP from minority ethnic groups in the UK or disabled young adults in care. Some groups for which we found no specific studies are represented by very small numbers of children, such as those in secure units (only 200 LACYP in England in 2007), and groups on which it is very difficult to conduct research or collect outcome data, such as those ‘missing or absent for more than 24 h’ (150 LACYP in England in 2007).

### Applicability of non-UK studies to UK care system and populations

The majority of studies included in this review were carried out in the USA, and applying the result of US studies may be problematic as the US foster care system differs from the UK system. Even within the USA, policies and practices vary significantly from state to state. For example, each state determines its own definition of maltreatment, its own laws based on federal regulations and its own level of investment in child welfare services. The organization of child welfare agencies also varies significantly across states. In some states, the child welfare system is administered at the state level, whereas in others it is administered at the county level (Bass *et al*. 2004). In one review, the authors noted in their discussion that social service and health provision is very different between the USA and the UK and that research findings from the USA cannot be uncritically applied to the UK (Holland *et al*. 2005).

Some European studies have been included in the review, from the Netherlands, Sweden, Finland and Norway, and non-European countries, Israel and Australia, which are known to have different policies and practices compared with the UK. It is not clear from the studies in this review how much these policies and practices differ, although one study states that Israel is different from other countries in that institutional placement is the most common solution for abused and neglected children (Shechory & Sommerfeld 2007). The issue of generalizability will need to be fully addressed if evidence from the international literature is used in the development of guidance for LACYP in the UK.

### Relationship of evidence to UK policy

The UK Government report ‘Every Child Matters: Change for Children’ sets out the national framework for local change programmes to build services around the needs of children and young people to maximize opportunity and minimize risk (Department for Education and Skills 2004). The Government's aim is for every child, whatever their background or their circumstances, to have the support they need to be healthy, stay safe, enjoy and achieve, make a positive contribution and to achieve economic well-being. The Department of Education has published regulations and guidance to improve the quality and consistency of care planning, placement and case review for looked-after children and to improve the care and support provided to care leavers. The full set of regulations and guidance will come into force on 1 April 2011 (Department for Education and Skills 2010). Some key factors identified in this review can be associated with a number of the outcomes from ‘Every Child Matters: Change for Children’ (Department for Education and Skills 2004). For example, ‘number of placements’ has been associated with adult employment, good health/mental well-being, risk of offending, covering the ‘being healthy’, ‘staying safe’, ‘making a positive contribution’ and ‘achieving economic well-being’ outcomes from ‘Every Child Matters: Change for Children’ (Department for Education and Skills 2004). Similarly, behavioural problems have been associated with sexual activity, referral for therapy and mental health service use, which relate directly to the ‘being healthy’ and ‘staying safe’ outcomes.

## Conclusion

This review represents an attempt to use the existing evidence base to map associations between potential risk factors, protective factors, interventions and outcomes for LACYP. The conceptual maps provide an overview of the key relationships addressed in the current literature that may usefully inform guidance development and highlight some potential gaps in terms of available evidence, in relation to understanding how interventions may reduce the otherwise very high risk of poor outcomes for this vulnerable group.

**Key messages**

- The mapping shows indirect as well as direct associations between factors, interventions and outcomes for looked-after children and young people, giving more information on the interplay between factors/interventions and outcomes.
- Placement stability and emotional and behavioural problems were identified as key mediators between underlying risk factors and outcomes.
- Three areas where sufficient evidence exists to conduct a systematic review are highlighted: support services for transition to adulthood, training and support for carers and improving access to services.
